# Declining Trends in Childhood TB Notifications and Profile of Notified Patients in the City of Harare, Zimbabwe, from 2009 to 2018

**DOI:** 10.1155/2020/4761051

**Published:** 2020-05-21

**Authors:** Phoebe Nzombe, Srinath Satyanarayana, Hannock Tweya, Collins Timire, Kelvin Charambira, Ronald T. Ncube, Christopher Zishiri, Riitta A. Dlodlo, Clemence Duri, Prosper Chonzi, Fredrick Mbiva, Nicholas Siziba, Charles Sandy

**Affiliations:** ^1^International Union Against Tuberculosis and Lung Disease, Harare, Zimbabwe; ^2^Centre for Operational Research, International Union Against Tuberculosis and Lung Disease, Paris, France; ^3^The Lighthouse Trust, Lilongwe, Malawi; ^4^International Union Against Tuberculosis and Lung Disease, Paris, France; ^5^City Health Department, City of Harare, Zimbabwe; ^6^Ministry of Health & Child Care, AIDS & TB Programme, Harare, Zimbabwe

## Abstract

Globally, childhood tuberculosis (TB among those aged <15 years) is a neglected component of national TB programmes in high TB burden countries. Zimbabwe, a country in southern Africa, is a high burden country for TB, TB-HIV, and drug-resistant TB. In this study, we assessed trends in annual childhood TB notifications in Harare (the capital of Zimbabwe) from 2009 to 2018 and the demographic, clinical profiles, and treatment outcomes of childhood TB patients notified from 2015–2017 by reviewing the national TB programme records and reports. Overall, there was a decline in the total number of TB patients (all ages) from 5,943 in 2009 to 2,831 in 2018. However, the number of childhood TB patients had declined exponentially 6-fold from 583 patients (117 per 100,000 children) in 2009 to 107 patients (18 per 100,000 children) in 2018. Of the 615 childhood TB patients notified between 2015 and 2017, 556 (89%) patient records were available. There were 53% males, 61% were aged <5 years, 92% were new TB patients, 85% had pulmonary TB, and 89% were treated for-drug sensitive TB, 3% for drug-resistant TB, and 40% were HIV positive (of whom 59% were on ART). Although 58% had successful treatment outcomes, the treatment outcomes of 40% were unknown (not recorded or not evaluated), indicating severe gaps in TB care. The disproportionate decline in childhood TB notifications could be due to the reduction in the TB burden among HIV positive individuals from the scale up of antiretroviral therapy and isoniazid preventive therapy. However, the country is experiencing economic challenges which could also contribute to the disproportionate decline in childhood TB notification and gaps in quality of care. There is an urgent need to understand the reasons for the declining trends and the gaps in care.

## 1. Introduction

Tuberculosis (TB) is an important cause of morbidity and mortality among children (aged <15 years) in low- and middle-income countries. World Health Organization (WHO) estimated that, in 2018, one million children developed TB, while 230,000 died due to the disease and 80% of these were under 5 years [1]. Studies from high TB burdened countries indicate that childhood TB accounts for 10% (range 5–15%) of all TB patients notified [2]. Early diagnosis and treatment are essential to reduce morbidity and mortality in children, but this is highly challenging in resource-limited settings.

The identification and diagnosis of TB in children is negatively affected by inadequate clinical expertise to diagnose and treat the disease resulting from high cost of in-service training [3]. This is worsened by the invasive procedures used to collect specimens such as nasopharyngeal and gastric aspirates. If a specimen is collected, healthcare workers face challenges in accessing diagnostic consumables and facilities. Therefore, specimens have to be transported to referral laboratories, and results can only be received after several days which can delay treatment initiation. In addition, lack of diagnostic equipment for extrapulmonary TB (EPTB) diagnosis limits access to prompt decision making as patients have to pay for services. Furthermore, nearly half of childhood TB patients are asymptomatic during early phases of the disease, and clinical features overlap with other diseases leading to late diagnosis, unfavourable treatment outcomes, and continued disease transmission [4].

WHO and its partners have developed a roadmap for reaching zero TB deaths among children by 2030 within the framework of the Global End TB Strategy [5]. Achieving this requires advocacy, commitment, financial resources, and joint effort from all stakeholders.

The primary responsibility for providing quality-assured childhood TB diagnosis and treatment services in high TB burdened countries rests with the national TB programmes (NTPs) [2]. NTPs are expected to formulate guidelines for management of childhood TB, build capacity of healthcare providers, and allocate adequate resources for establishing diagnostic and treatment services. They also have a responsibility to include childhood TB data in routine recording and reporting systems, periodically review childhood TB data, and undertake operational research to determine barriers and solutions for providing optimal care [2, 6]. Globally, NTPs of high TB burdened countries are unable to implement all these recommendations commensurate to the need [7].

Zimbabwe is a low-income country in Southern Africa with a high TB and HIV burden [8]. The annual number and proportion of childhood TB patients notified under the Zimbabwe NTP declined from 4,596 patients (10% of the total TB patients) in 2010 to 1,543 patients (6% of the total TB patients) in 2018 [9, 10]. The NTP has been implementing a National TB Strategic Plan (2017–2020), and one of its key activities is to strengthen childhood TB case finding efforts and increase the proportion of childhood TB patients from 7% in 2016 to 12% by 2020 [11].

It is unknown whether implementation of the strategic plan has halted and/or reversed the long-term declining trends in the number and proportion of childhood TB patients in Zimbabwe. In addition, the sociodemographic and clinical profiles, management, and treatment outcomes of children with TB in Zimbabwe have not been studied before, and therefore the gaps in providing care (subsequent to TB diagnosis) are unknown.

Given this paucity of information on childhood TB in Zimbabwe, we conducted an operational research study with an aim to describe the trends, profile, management, and treatment outcomes of childhood TB patients notified under the NTP in the city of Harare. Our study had two specific objectives: to describe the annual trends in number, proportion, and rates of childhood TB patients notified from 2009 to 2018 and to describe the demographic, clinical characteristics, and treatment outcomes of childhood TB patients notified from 2015 to 2017.

## 2. Materials and Methods

### 2.1. Study Design

For the first objective, we used an ecological study design, and for the second objective, we used a cohort study design. We used secondary data routinely recorded and reported by the NTP for both objectives.

### 2.2. Study Setting

#### 2.2.1. General Setting

Zimbabwe is a landlocked, southern African country, which is among the 30 high burden countries for TB, TB/HIV, and multidrug-resistant TB (MDR-TB)/rifampicin-resistant TB (RR-TB) [11]. Two-thirds (63%) of notified TB patients in the country in 2018 were coinfected with HIV [1]. TB mortality rate among HIV-negative population has declined 33%, from 18 per 100,000 population in the year 2000 to 7.7 per 100,000 population in 2018 [1]. TB mortality rate among HIV-positive population has significantly declined from a peak of 158 per 100,000 in 2006 to 24 per 100,000 population in 2018 due to massive scale up of antiretroviral therapy (ART) services [1].

#### 2.2.2. Zimbabwe NTP

At the national level, the NTP coordinates policy formulation, resource mobilization, and implementation of TB care and prevention activities. TB interventions are guided by the National TB Strategic Plan (2017–2020) which is aligned to the National Health Strategy (2016–2020) as well as the Global End TB strategy [5, 11]. The district is the lowest programme management unit. The day-to-day coordination of programme implementation is the responsibility of the district and provincial TB coordinators [12]. The NTP has a standard recording and reporting system, which is predominantly paper-based at the primary health facility, and efforts are underway to roll out a patient-based electronic surveillance system. The operational definition for classifying patients, their treatment outcomes, and recording and reporting system are aligned with those recommended by the WHO for NTPs [12].

In the public sector, TB diagnostic services are integrated into the primary healthcare system and are offered free-of-cost to the patients (both children and adults). Pulmonary TB diagnosis for children at the primary health facility is established through a combination of tuberculin skin test and Xpert MTB/Rif® test conducted on gastric washings or sputum. Sputum smear microscopy is conducted in the absence of Xpert MTB/Rif ® test [13]. Diagnosis of clinical or EPTB is made by a medical doctor at a district hospital. Upon TB diagnosis, all children are registered with a unique identification number and are initiated on standard treatment according to their weight as per the National TB Guidelines (Table 1) [14]. The NTP also collaborates with the private sector which can diagnose TB in both adults and children, however, upon diagnosis, all patients have to be referred to public health facilities for treatment [15].

In addition, all children diagnosed with TB are tested for HIV (if previously HIV negative or unknown). All HIV-positive children are linked to ART services. The TB treatment regimens for HIV-positive and HIV-negative children are similar. The compliance to anti-TB treatment is ensured through a family caregiver [13].

#### 2.2.3. Harare

Harare, the capital city of Zimbabwe, is divided into eight districts and in 2018 had a projected population of 1.5 million [16]. The Ministry of Health and Child Care provides health services in the city through the City Health Department which is responsible for the operation of the two infectious disease hospitals, 12 polyclinics (three clinics in one with Maternity Unit, Primary Care Clinic and Family health services clinic), seven primary care clinics, 15 satellite clinics, and six family health service clinics. Childhood TB diagnostic and management services are offered in all of these facilities as per the NTP guidelines [17].

### 2.3. Study Population, Sample Selection, and Sample Size

For objective 1, we used data of all TB patients diagnosed in the city of Harare disaggregated by age group from 2009 to 2018.

For objective 2, all children (0–14 years) diagnosed with any form of TB in the city of Harare from January 2015–December 2017 were included in this study (without any exclusion). The study included children with drug-sensitive TB as well as drug-resistant TB.

### 2.4. Data Variables, Sources of Data, and Data Collection Form

For objective 1, the data variables included yearwise total number of all TB patients and number of childhood TB patients registered (disaggregated by age (0–4 years, 5–14 years, and gender), and corresponding estimated midyear population of 0–14 years). The source of data was province annual/quarterly TB case finding reports obtained from the provincial TB office and the estimated midyear population of 0–14 years from city of Harare population records. The data were collected on a structured proforma by the principal investigator.

For objective 2, the data variables included: TB number, age, gender, type of residential area, HIV status, ART status (if HIV positive), type of TB (new, retreatment, and MDR), disease classification (pulmonary/extra-pulmonary), treatment regimen, and TB treatment outcome (treatment completed/cured, lost to follow-up, death, failure, transferred out, and not evaluated). All TB-related definitions were according to the WHO definitions and reporting framework for tuberculosis—2013 revision) [18]. The source of data was from the district TB register and facility treatment registers. Data were collected into a paper-based structured data collection form.

### 2.5. Analysis and Statistics

Data were double entered from the structured paper-based data collection form into EpiData entry client (Version 4.4.3.1, EpiData Association, Odense, Denmark).

For objective 1, we conducted a trend analysis of the following childhood TB notification measures: annual number of childhood TB patients notified; proportion of childhood TB patients among all TB patients notified; and annual childhood TB notification rates (number of TB patients per 100,000 persons aged 0–14 years). Linear and nonlinear regression models were used to assess the trends. We assessed for model fit using goodness of fit (*R*^2^) values, and the best fit model (linear or nonlinear) was used for making inferences on the trends. For objective 2, we summarized the demographic, clinical characteristics, diagnostic tests, treatment regimens, and treatment outcomes in numbers and proportions. Since a large proportion of childhood TB patients' treatment outcomes were unknown, we refrained from assessing the association between demographic and clinical characteristics with the treatment outcomes. All data analysis were conducted using Microsoft Excel and STATA (Version 15, Stata Corporation, Texas, USA).

### 2.6. Ethics

We obtained ethical approval from the Medical Research Council of Zimbabwe (approval number MRCZ/E/256) and from the Ethics Advisory Group of The Union, Paris, France (approval number 46/19).

## 3. Results

**Figure 1:**
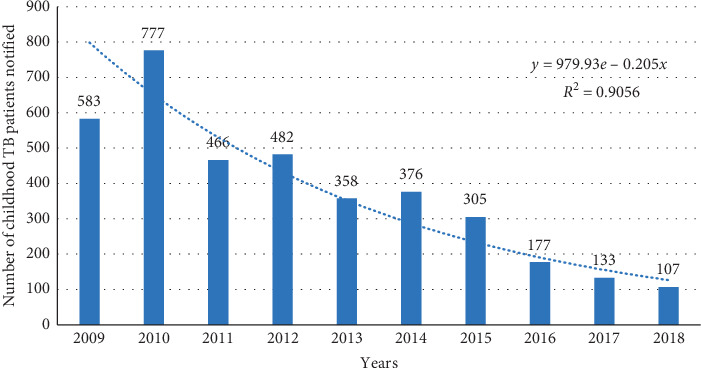
Trends in annual number of childhood TB notifications in Harare, Zimbabwe, 2009–2018.

**Figure 2:**
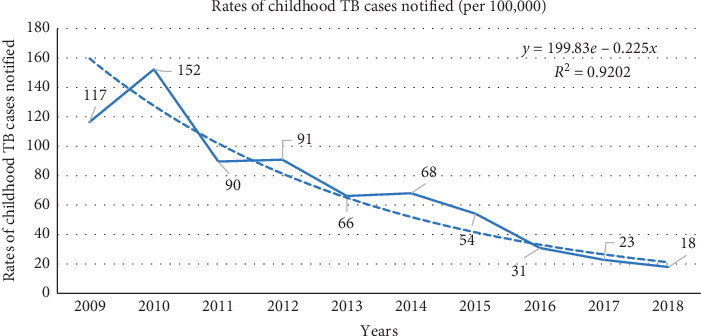
Trends in annual childhood TB notification rate (cases per 100,000 persons aged <15 years) in Harare, Zimbabwe, 2009–2018.

Figures 1 and 2 show trends in annual number of childhood TB patients notified and childhood TB notification rate (cases per 100,000 children) in Harare, Zimbabwe, between 2009 and 2018, respectively. The annual number of TB patients notified (and the annual case notification rate) declined exponentially from 583 patients (117 cases per 100,000 children) in 2009 to 107 patients (18 cases per 100,000 children) in 2018.

**Figure 3:**
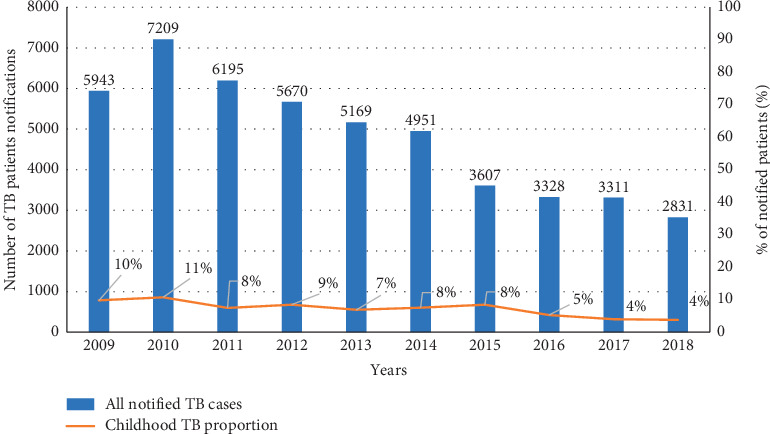
Trends in annual total number of patients notified and childhood TB as a proportion of total patients notified in Harare, Zimbabwe, 2009–2018.

The trends in annual total number of TB patients notified and childhood TB as a proportion of total TB patients notified in the city of Harare from 2009 to 2018 are given in Figure 3. Total number of TB patients notified declined from 5,943 in 2009 to 2,831 in 2018. The proportion of childhood TB patients reduced from 10% to 4% during the same period.

A total of 615 childhood TB patients were notified between 2015 and 2017. Of these, 556 (89%) patient records were available, and their demographic and clinical characteristics are given in Table 2. There were 53% males, 61% were aged <5 years, 92% were new TB patients, 85% had pulmonary TB, and 89% were treated for drug-sensitive TB. Eighty-five percentage of the children had a known HIV status. About 40% were HIV positive, and of these, 59% were on ART. Their treatment outcomes disaggregated by the type of TB regimen as outlined in Table 3. Overall, 58% had successful treatment outcome, 5% had unsuccessful outcome, and 37% did not have a treatment outcome recorded.

## 4. Discussion

This is the first study to assess trends in childhood TB notifications and describe the profile of childhood TB patients in Zimbabwe. The study findings showed a general decrease in childhood TB notifications and childhood TB notification rates. Only 59% of the HIV-positive children were on ART, and 3% of the children had drug-resistant TB. The study also showed low treatment success rates of 58%. These findings highlight challenges and provide insights into strategies that may improve delivery of services to children with TB.

There was a 6-fold decline in the number of childhood TB patients in Harare over the last ten years, and the decline was steeper in children than in adults. The declining trends are similar to findings in Zambia from 2004–2010 [19], and in Kampala (Uganda) where the city recorded a decline in childhood TB notifications between 2011 and 2015 [20]. However, the declining trend is in contrast to the increasing trends documented in Nigeria, South Africa, and Malawi which all recorded an increase in childhood TB notifications [21–23]. Zimbabwe is a high HIV burden setting with rapid scale up of ART services. A Zimbabwean study by Takarinda et al. indicated that, in a high HIV burden setting, scale up of ART services can result in a decline in the burden of HIV which, in turn, could have a huge impact on TB incidence and mortality [24].

However, the declining trend may not be a true reflection of the disease burden because a study conducted in Harare in 2016 showed that 29% of children in the city were stunted [25]. This means that a significant proportion of Harare children are at a high risk of developing TB disease if exposed to infection because malnutrition can result in secondary immunodeficiency which increases a child's susceptibility to TB infection. It is possible that children suffering from TB in the city could actually be on the increase as a result of malnutrition, but they are not being diagnosed by the health delivery system. The fact that children with TB are being missed was demonstrated by the International Union against Tuberculosis and Lung Disease Zimbabwe (through the Challenge TB project) which supported the implementation of a Childhood TB intervention package in Makoni district with low childhood TB notification rates. After implementation of the intervention, the number of children notified increased from 3% to 6% which showed that the health delivery system was failing to diagnose children suffering from TB [26].

Studies from South Africa have shown that childhood TB notifications are higher in crowded environments where children spend extended periods of time with adults suffering from TB [4, 27]. Living conditions in these settings are characterised by poverty, tobacco smoking, alcohol, and substance abuse. When exposed to TB, children living in this environment have a higher chance of infection and development of active TB disease [4, 28]. Similar conditions are prevalent in and around the city which, according to the Harare Slum Profiles Report, has at least 60 informal settlements characterised by poor living conditions [29]. This means that there are many children living in these settlements who could be suffering from TB but do not have access to healthcare and are not identified and linked to care. And therefore, we strongly believe that the decline in notifications is not reflective of the true situation but is due to the health systems' failure to detect and treat childhood TB.

Two versions of the Zimbabwe National TB Guidelines (2010 and 2017) were released in the lifespan of the study. However, the decline in TB notifications cannot be attributed to differences in case definitions of children with presumptive TB outlined in these guidelines. The 2010 guidelines state that a child with presumptive TB is defined as any child, who is a TB contact of an adult or older child, has chronic cough or wheeze for more than two weeks after receiving antibiotic or other appropriate treatment, loss of weight or failure to gain, persistent fever (>38˚C) for greater than two weeks, and has enlarged lymph nodes; if HIV positive, the child should be screened at every visit to a health facility [30]. The 2017 guidelines are similar, but the only difference is that the cough duration is reduced from two to one week. The reduction in the duration of the cough was supposed to increase the number of children diagnosed of TB since more children were eligible to be tested for the disease [14].

The profile of childhood TB patients indicates that more than half (60%) of them were in the 0–5 years age group which is similar to findings from South Africa, Zambia, and Malawi [19, 21, 22]. This is because children in this age group have an undeveloped immune system; therefore, they are at a higher risk of developing active TB if exposed and infected with TB. Furthermore, children in this age group spend prolonged periods of time in close proximity to their caregivers; therefore, they are at a high risk of contracting TB if their caregiver is suffering from the disease [28]. Nearly half of the children (48%) were clinically diagnosed and had not undergone a sputum test. This is in line with studies from Malawi and Ethiopia [3, 21]. This is because children have difficulty in producing sputum, and some health workers working at lower levels lack clinical expertise to collect appropriate biological specimens such as gastric aspirates [13]. In our study, only 15% of the childhood TB patients had EPTB which is similar to findings documented in South Africa, Zambia, and Malawi where they recorded 13%, 6%, and 15% EPTB, respectively [31, 32]. This is, however, in contrast to other studies conducted in the Democratic Republic of Congo and Nigeria which found higher proportions of EPTB (56% and 42%, respectively) [31, 33]. The low proportion of children diagnosed with EPTB could indicate challenges in diagnosis of EPTB patients in Harare where only a medical doctor who is usually based at a hospital can make such a diagnosis.

Only 59% of the HIV-positive children in our study were on ART which is in contrast to studies in Malawi and Kenya where 75% and 92% of the HIV-positive children were enrolled on ART, respectively [34, 35]. This finding in our study is a cause for concern as the Zimbabwean National TB guidelines recommend universal access to ART for all HIV-positive TB patients. However, this finding could be as a result of poor recording and reporting as national reports state that 76% of HIV-positive children in Zimbabwe were on ART [36]. This could mean that more children in Harare could actually be on ART, but this was not documented in the facility TB registers.

Another area of concern is the presence of DR-TB (3% of the study participants) among the childhood TB patients, which most likely indicates primary transmission of the disease. This differs from an Ethiopian study where only 0.4% of their childhood TB patients had DR-TB [37]. However, our findings were lower than statistics documented in South Africa where 11.3% of their childhood TB patients had DR-TB [38].

The treatment outcomes of 37% of the childhood TB patients in our study were not recorded/unknown which indicates deficiencies in recording and reporting and in provision of quality TB treatment services to children. This is similar to findings of a study conducted in Malawi where 21% of the childhood TB patients had unknown treatment outcomes [39]. The poor recording and reporting identified by this study could be as a result of staffing challenges in the health sector. The United Nations Development Programme reported that Zimbabwe has lost a significant number of skilled health workers and has experienced deterioration of its health infrastructure [40]. The city of Harare has not been spared from this brain drain, and the situation is worse for the local authority as it has battled several disease outbreaks such as the typhoid outbreak in 2012 and cholera outbreaks in 2018 which negatively impacted the performance of public health programmes (including TB) as health workers had to be deployed to the affected areas to contain the outbreaks [41, 42]. These findings are comparable with findings from Ethiopia and Uganda where poor quality of care in TB patient management was attributed to low health worker motivation [43]. However, investigations into what happened to the childhood TB patients whose outcomes were not recorded is an area that could be considered for future research.

Our study findings should be viewed with the following limitations. First, we used routine data, and some information was missing from the facility TB registers. Information on outcomes was not available in 37% of the children, and the findings on treatment outcomes may not be extrapolated to the entire population of children with TB. Second, health facilities in Harare have relatively better health infrastructure, which may lead to better care and support for childhood TB than in the other parts of the country. Despite these limitations, we believe that use of routine data reflects performance of the TB programme in Harare City, and the findings are useful in providing information on the delivery of TB services in children in Zimbabwe and other comparable settings.

In conclusion, childhood TB notifications have declined 6-fold in the last ten years in Harare, and the profile and treatment outcomes of childhood TB patients indicate several gaps in the provision of care. There is an urgent need for health system strengthening to improve the situation.

## Figures and Tables

**Table 1:** Tuberculosis treatment regimens for childhood TB in Zimbabwe.

Weight bands	Intensive phase^*∗*^		Continuation phase
	RHZ^*∗∗*^ (75 mg/50 mg/150 mg)	E (100 mg)	RH (75 mg/50 mg)
4–7.9 kg	1	1	1
8–11.9 kg	2	2	2
12–15.9 kg	3	3	3
16–24.9 kg	4	4	4
25 kg and above	Use adult formulations		

^*∗*^Number of tablets. ^*∗∗*^Recommended dose of TB medicines by weight bands using Fixed Dose Combinations. H = isoniazid; R = rifampicin, Z = pyrazinamide; E = ethambutol.

**Table 2:** Demographic and clinical characteristics of childhood TB patients in Harare, Zimbabwe, 2015–2017.

Characteristics	*N*	(%)
Total	556	(100)

Gender		
Male	292	(53)
Female	264	(47)

Age (years)		
<5	339	(61)
5 to 9	106	(19)
10 to 14	109	(20)
Not recorded	2	(0)

Type of TB patient		
New	509	(92)
Retreatment	17	(3)
DR-TB	18	(3)
Not recorded	12	(2)

Type of TB		
Pulmonary TB	474	(85)
Extrapulmonary TB	75	(13)
Not recorded	7	(1)
HIV status		
Negative	291	(52)
Positive	222	(40)
Unknown	14	(3)
Not recorded	29	(5)

ART status (*n* = 222)		
On ART	131	(59)
Not on ART	11	(5)
Not recorded	80	(36)

Sputum smear		
Negative	164	(29)
Positive	52	(9)
Not done	268	(48)
Not recorded	72	(13)
Treatment regimen		
Drug-sensitive	497	(89)
DR-TB	18	(3)
Not recorded	41	(7)

**Table 3:** Treatment outcomes of childhood TB patients enrolled for TB treatment in Harare, Zimbabwe, 2015–2017, disaggregated by the type of TB treatment regimen.

Treatment outcome	Drug-sensitive TB regimen (*N* = 497)		Drug-resistant TB regimen (*N* = 18)		Unknown (*N* = 41)		Total	
	*N*	%	*N*	%	*N*	%	*N*	%
Favourable								
Cured	30	(6)	3	(17)	2	(5)	35	(6)
Completed	258	(52)	8	(44)	24	(59)	290	(52)

Unfavourable								
Died	1	(0)	0	(0)	0	(0)	1	(0)
Lost to follow-up	1	(0)	0	(0)	0	(0)	1	(0)
Transferred out	7	(1)	0	(0)	1	(2)	8	(1)
Not evaluated	17	(3)	0	(0)	1	(2)	18	(3)

Not recorded/unknown	183	(37)	7	(39)	13	(32)	203	(37)

## Data Availability

The data used to support the findings of this study are available from the corresponding author upon request.
